# Replication Stress Induces Micronuclei Comprising of Aggregated DNA Double-Strand Breaks

**DOI:** 10.1371/journal.pone.0018618

**Published:** 2011-04-15

**Authors:** Bing Xu, Zhaoliang Sun, Zhaojian Liu, Haiyang Guo, Qiao Liu, Haiyan Jiang, Yongxin Zou, Yaoqin Gong, Jay A. Tischfield, Changshun Shao

**Affiliations:** 1 Key Laboratory of Experimental Teratology, Ministry of Education, and Institute of Molecular Medicine and Genetics, Shandong University School of Medicine, Jinan, Shandong, China; 2 Department of Genetics, Rutgers University, Piscataway, New Jersey, United States of America; Texas A&M University, United States of America

## Abstract

**Background:**

Micronuclei (MN) in mammalian cells serve as a reliable biomarker of genomic instability and genotoxic exposure. Elevation of MN is commonly observed in cells bearing intrinsic genomic instability and in normal cells exposed to genotoxic agents. DNA double-strand breaks are marked by phosphorylation of H2AX at serine 139 (γ-H2AX). One subclass of MN contains massive and uniform γ-H2AX signals. This study tested whether this subclass of MN can be induced by replication stress.

**Principal Findings:**

We observed that a large proportion of MN, from 20% to nearly 50%, showed uniform staining by antibodies against γ-H2AX, a marker of DNA double-strand breaks (DSBs). Such micronuclei were designated as MN-γ–H2AX (+). We showed that such MN can be induced by chemicals that are known to cause DNA replication stress and S phase arrest. Hydroxyurea, aphidicolin and thymidine could all significantly induce MN-γ–H2AX (+), which were formed during S phase and appeared to be derived from aggregation of DSBs. MN-γ–H2AX (−), MN that were devoid of uniform γ-H2AX signals, were induced to a lesser extent in terms of fold change. Paclitaxel, which inhibits the disassembly of microtubules, only induced MN-γ–H2AX (−). The frequency of MN-γ–H2AX (+), but not that of MN-γ–H2AX (−), was also significantly increased in cells that experience S phase prolongation due to depletion of cell cycle regulator CUL4B. Depletion of replication protein A1 (RPA1) by RNA interference resulted in an elevation of both MN-γ–H2AX (+) and MN-γ–H2AX (−).

**Conclusions/Significance:**

A subclass of MN, MN-γ–H2AX (+), can be preferentially induced by replication stress. Classification of MN according to their γ-H2AX status may provide a more refined evaluation of intrinsic genomic instabilities and the various environmental genotoxicants.

## Introduction

Scoring of micronuclei (MN) is widely used to monitor genomic instability and genotoxic exposure [1]–[3]. Elevation of MN is commonly observed in cells bearing intrinsic genomic instability and in cells exposed to genotoxic agents. Compared to assays for other cytogenetic biomarkers, such as chromosomal aberrations and sister chromatid exchanges (SCE), the MN assay is simpler and less time consuming. Because MN assay allows the analysis of much larger samples than other assays, it is also more sensitive. With the development of a flow cytometry based assay, MN can be scored on tens of thousands of peripheral blood erythrocytes in terms of minutes [4], making it possible to evaluate mutagens and genetic conditions that only cause subtle increase in genomic instability.

MN can be divided into C+ MN and C- MN based on the presence or absence of centromere(s). The presence of centromeres in MN, C+ MN, indicates their origin from whole chromosomes. C- MN are presumably formed from acentric chromosome fragments. Based on their ability to induce C+ MN and C- MN, respectively, mutagens have accordingly been divided into aneugens and clastogens [3]. Regardless of their origins, both types of MN are formed in anaphase when chromosome fragments or whole chromosomes fail to segregate into the daughter cells. A recent live cell imaging study showed that MN induced by mitomcycin C, γ-rays and vincristine were all formed during late phases of mitosis [5]. However, MN were also reported to form during interphase, due to disruptions in chromatin remodeling [6], [7], or oncogene amplification [8].

Characterization of the DNA contents in MN by chromosome painting revealed that not all chromosomes are equally represented in MN. For example, human chromosomes 9, X and Y are overrepresented in the MN of cultured lymphocytes, while chromosome 12 is underrepresented [9]. In cultured human lymphocytes, the frequency of C+ MN is found to increase with aging, due to an age-dependent micronucleation of the X and Y chromosomes [3]. While the frequency of MN increases with exposure to mutagens or with aging, various genetic conditions can also lead to an elevation of spontaneous frequency of MN. For example, cells heterozygous for *BubR1*, a spindle checkpoint gene, exhibit a higher frequency of MN, presumably formed from chromosome laggards [10]. Deficiency in each of several DNA repair pathways is associated with increased frequency of MN [11]–[13]. Because MN may be formed by different mechanisms in different conditions, subclassification of MN, when possible, in each experimental condition may help gain more insight into the mechanisms by which MN form.

It was recently reported that massive and uniform phosphorylation of H2AX characterizes a subclass of MN in cultured mammalian cells [14]. Phosphorylation of H2AX, or γ-H2AX, marks the presence of double-strand breaks (DSBs) in DNA. The formation of DSBs is accompanied by a rapid phosphorylation of histone H2AX at serine 139 in chromatin domains surrounding DSBs [15], [16]. It is believed that phosphorylation of H2AX plays a critical role in recruiting DNA repair factors to the damaged site and facilitate DNA repair [17], [18]. With antibodies against the phosphorylated form of H2AX (γ-H2AX), DSBs can be easily visualized and quantified under a microscope. Discrete nuclear γ-H2AX foci have become a sensitive and reliable marker of DSBs [19]. In addition to their occurrence as discrete nuclear foci, γ-H2AX signals can occupy a whole nucleus in a diffuse and uniform manner in cells experiencing replication stress [20] in cells exposed to high concentration of NaCl [21] or in cells exposed to hypoxia [22]. γ-H2AX also marks sex bodies uniformly from late zygotene, throughout pachytene, to early diplotene during spermatogenesis [23] and probably plays a role in silencing unsynapsed chromosomes [24].

In this report we studied whether the formation of MN with uniform labeling of γ-H2AX, designated as MN-γ-H2AX (+), is associated with DNA replication stress. By scoring the MN in cells treated with agents that cause replication stress and in cells that are defective in carrying out DNA replication, we demonstrated that the formation of MN-γ-H2AX (+) is associated with DNA replication stress.

## Materials and Methods

### Cell culture

MCF-7, PC3 and LLC cells were obtained from Shanghai Cell Bank, Chinese Academy of Sciences. HEK-293 cells and normal human dermal fibroblast (NHDF) cells were obtained from the Institute of Basic Medical Sciences, Chinese Academy of Medical Sciences (Beijing). U2OS cells were as described [25]. The immortalized cells were maintained in Dulbecco's modified Eagle's medium (DMEM) supplemented with 10% fetal bovine serum (FBS), 100 µg/ml penicillin and 100 µg/ml streptomycin. Normal human dermal fibroblast (NHDF) cells were maintained in DMEM/F12 containing 10% FBS, 1 mM L-glutamine (sigma), and 4 ng/ml bFGF (PeproTech). Primary mouse skin (ear) fibroblasts (MSF) were prepared as described [26]. All cells were cultured in a humidified atmosphere of 5% CO2 at 37°C. Cells were grown on coverslips in 6-well plates before different treatments. Hydroxyurea, aphidicolin, and thymidine were purchased from Sigma Chemical Co (St. Louis, MO). Paclitaxel was from Sangon Biotech.

### Immunofluorescence

Antibodies against phospho-H2AX (Ser139) (DAM1661039) was purchased from Millipore (Billerica, MA). TRITC-conjugated Goat anti-mouse secondary antibody was from Jackson Immuno Research Laboratories. Cells, after being washed in PBS, were fixed in 4% paraformaldehyde for 15 min, washed three times in PBS and permeabilized for 10 min in 0.2% Triton X-100, and then blocked in 10% normal goat serum overnight at 4°C. After washed in PBS, the coverslips were incubated with anti-phospho-H2AX antibody overnight at 4°C. Cells were then washed in PBS, and incubated with TRITC-conjugated secondary antibody for 1 h at room temperature. Cells were washed in PBS for three times and counterstained with 4, 6-diamidino-2-phenylindole (DAPI). The coverslips were mounted on slides for examination.

### Cell cycle analysis by flow cytometry

Analysis of cell cycle distribution was carried out by flow cytometry. Cells were washed with PBS, trypsinized and then collected. The cells were fixed in 70% ice-cold ethanol at −20°C overnight. After washed with PBS, cells were gradually resuspended in PBS containing 2.5 µg/ml RNase, 0.2% Tween20 and 5 µg/ml propidium iodide (PI) for 30 min at 37°C. Cell cycle distribution was analyzed by measuring DNA content using a FACSCalibur flow cytometer (Beckman Coulter).

### BrdU incorporation assay

DNA synthesis was studied by pulse labeling cells with 50 µM of Bromodeoxyuridine (BrdU) for 30 min. Cells were washed twice in PBS, fixed in 4% paraformaldehyde for 20 min. They were then washed three times in PBS, permeabilized in 0.2% Triton X-100 for 20 min, denatured in 2 M HCl for 20 min, and neutralized in 0.1 M sodium borate for 15 min. Afterwards, coverslips were blocked in 5% normal goat serum for 1 h and then incubated with anti-BrdU antibodies (Santa Cruz Biotechnology) overnight at 4°C. Cells were then washed three times in PBS, incubated with TRITC-conjugated secondary antibodies for 1 h at room temperature. And then cells were washed in PBS for three times and counterstained with DAPI. The coverslips were mounted on slides with anti-fading medium. The samples were examined with a fluorescence microscope (Olympus, DP71) and all experiments were repeated three times.

### RNA interference of RPA1

An oligonucleotide specifically against human *RPA1* (5′-CTGGTTGACGAAAGTGGTG-3′) was inserted into the BglII/HindIII sites of the pSUPER.neo+GFP vector (OligoEngine) according to the manufacturer's instructions. The construct was verified by DNA sequencing.

For stable transfection, MCF-7 cells were maintained in culture medium free of antibiotics at a density of 8×10^4^ cells/well in a 24-well plate. After overnight incubation, the construction containing RPA1-shRNA (shRPA1) and control (shNeg) were introduced into cells using Lipofectamine 2000 reagent (Invitrogen) according to the manufacturer's instructions. Cells were incubated in the medium (containing selection drug) 24 h after transfection. Stably transfected cells, after incubated in selective medium for 3–4 weeks, were tested by real-time quantitative RT-PCR assay and western blotting analysis, and then used for further study.

### RNA Isolation and Real-time quantitative RT-PCR assay

Total RNA was isolated using Trizol reagent (Invitrogen) according to the manufacturer's introduction. Any potential DNA contamination was removed by RNase-free DNase treatment. cDNA was obtained by reverse transcription-PCR of 1 µg of total RNA using the AMV reverse transcriptase (Promega).

Real-time quantitative RT-PCR was performed using the SYBR green PCR Master Mix (Applied Biosystems) in an ABI Prism 7500 sequence detection system (Applied Biosystems). Human GAPDH gene was used as control. The primers for SYBR were from primer bank of Harvard Medical School (http://pga.mgh.harvard.edu/primerbank/). Primer sequences for RPA1 and GAPDH are described as follow:

RPA1

5′-TCCAGTGCCCTATAATGAAGGA-3′ (Forward)
5′-GTAGAACCCATTCCCGAGCTT-3′ (Reverse)

GAPDH

5′-CAGAACATCATCCCTGCCTCTAC-3′ (Forward)
5′-TTGAAGTCAGAGGAGACCACCTG-3′ (Reverse)

### Western blotting analysis

Cells were harvested and lysed with cell lysis buffer for Western and IP (Beyotime) according to the manufacturer's instructions. Protein concentration was determined with BCA Protein Assay kit (Beyotime) using BSA as a standard. Protein samples were subjected to SDS-PAGE (12%) and transferred electrophoretically to PVDF membranes. After blocking with 5% skimmed milk, the membrane was incubated with specific primary antibodies overnight at 4°C. Mouse anti-human RPA1 (sc-28304) antibodies was from Santa Cruz Biotechnology and β-actin was from Sigma. Proteins of interest were detected with horseradish peroxidase-conjugated secondary antibody for 1 h and visualized by ECL Western Blotting Substrate (Thermo Scientific).

### Scoring of MN

**Figure 1:**
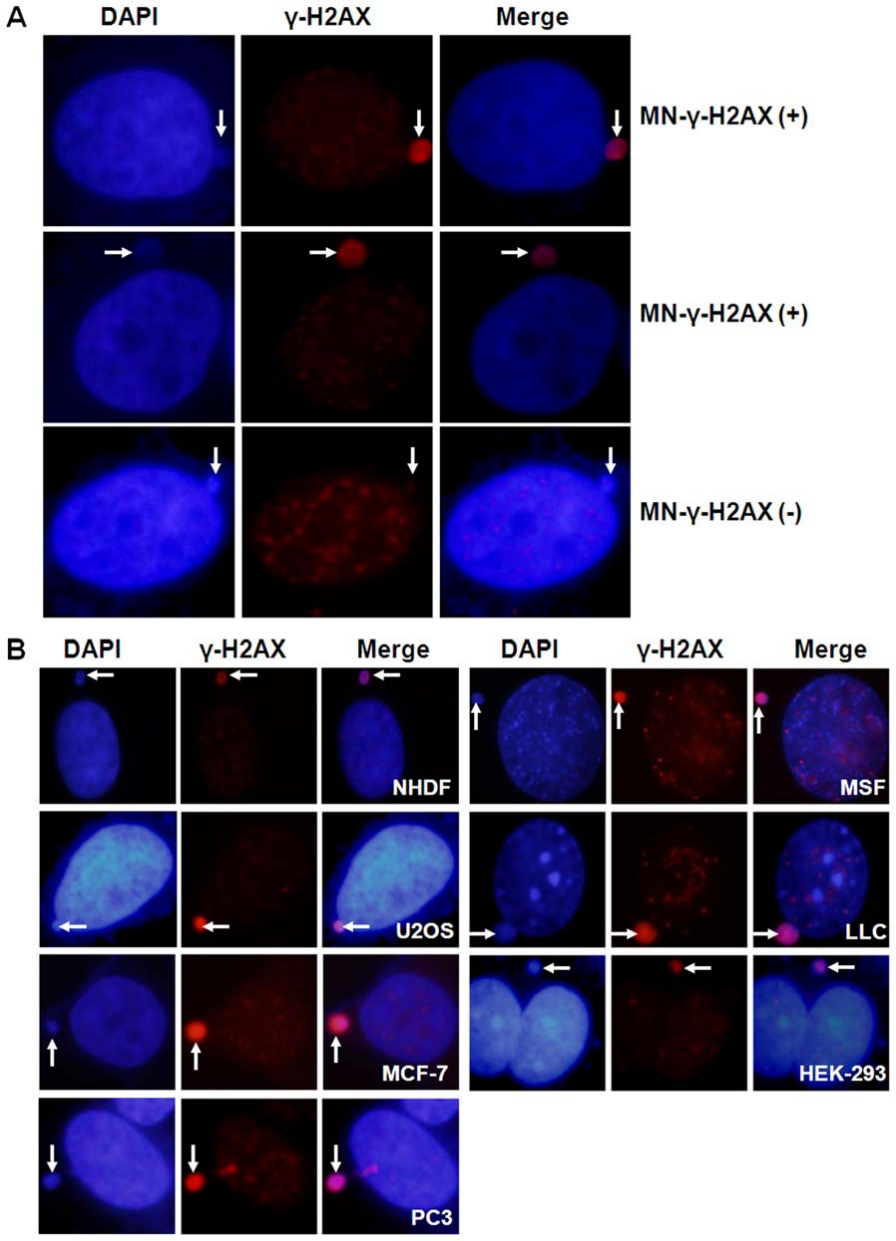
MN-γ–H2AX (+) in mammalian cells. A. Examples of MN-γ–H2AX (+) and MN-γ–H2AX (−) in MCF-7 cells. B. MN-γ-H2AX (+) in various mammalian cell lines. Cells were grown on coverslips in 6-well plates 24 h before they were processed for immunofluorescence staining with anti γ–H2AX antibody.

The samples were coded and examined with an Olympus DP71 fluorescence microscope. Nuclei were scored first for MN by their DAPI staining, using excitation filter BP330-385 and barrier filter BA420, under a 100× objective. MN were then examined for the presence or absence of **γ**-H2AX signals (excitation filter BP510-550, barrier filter BA590). A MN is designated as MN-**γ**-H2AX (+) if it is uniformly and more intensively stained than the nucleus (Figure 1). Cells with three or more MN were not included to avoid possible artifacts due to catastrophic cellular events.

### Statistical analysis

Data were mean ± s.d. from three independent experiments. The statistical significance of the differences in micronuclei data was analyzed using the Chi-square Pearson test. All the other data were analyzed using student's t test. *P* value<0.05 was taken as statistically significant.

## Results

### Presence of γ-H2AX in MN in mammalian cells

Micronuclei occur spontaneously at the frequency of 2 to 15×10^−2^ in cultured mammalian cells kept in normal growth condition (Table 1). While most of micronuclei were observed as single MN in their cells, occasional cells contain two or more MN. Cells with three or more MN were not considered in the frequency calculation.

**Table 1:** Occurrence of MN-γ–H2AX (+) in mammalian cells.

Cell line	No. Cells	No.MN	MN (×10^−2^)	No.MN-γ-H2AX(+)	MN-γ-H2AX(+)/MN(%)	MN-γ-H2AX(+) (×10^−2^)
HEK-293	1011	72	7.12	22	30.56	2.18
NHDF	2033	30	1.48	7	23.33	0.34
U2OS	1002	46	4.59	13	28.26	1.30
MCF-7	1009	63	6.24	14	22.22	1.39
PC3	1034	17	1.64	8	47.06	0.77
MSF(1^st^ pass)	1026	25	2.44	6	24.00	0.58
MSF(5^th^ pass)	1002	150	14.97	31	20.67	3.09
LLC	1001	143	14.29	41	28.67	4.10

NHDF, Normal Human Dermal Fibroblasts; MSF, Primary mouse skin (ear) fibroblasts.

MN were divided into two classes by the presence or absence of **γ-**H2AX in the MN. **γ**-H2AX signals, if present, usually occupies the majority or the whole micronucleus and usually displayed a diffuse, and often uniform, pattern throughout the whole MN. Very rarely were they observed to manifest as discrete **γ**-H2AX foci. We designated those MN coated with **γ**-H2AX signals for at least half of the whole MN as MN-**γ-**H2AX (+) (Figure 1A), and the others as MN-**γ-**H2AX (−). The multiple MN derived from a single cell are usually not uniformly negative or positive for **γ**-H2AX, suggesting that MN-**γ-**H2AX (+) do not form from the fragmentation of a nucleus with global H2AX phosphorylation.

The spontaneous frequencies of MN-**γ-**H2AX (+) vary among different cell lines, in the range from <1×10^−2^ to 4×10^−2^ (Table 1). In some cell lines, the MN-**γ-**H2AX (+) accounted for nearly half of the total MN. Interestingly, with increasing passage number, the frequency of MN-**γ-**H2AX (+) in mouse skin fibroblasts increased (Table 1).

### Induction of MN-γ-H2AX (+) by agents that cause DNA replication stress

Phosphorylation of H2AX usually marks double-strand breaks (DSBs) and stalled replication forks. In cells experiencing replication stress γ-H2AX signals occupy a whole nucleus in a diffuse and uniform manner [20]. The diffuse and uniform manner of γ-H2AX signals in MN-γ-H2AX (+) resembles the distribution pattern of γ-H2AX signals in nuclei of cells experiencing replication stress. We speculated that the formation of MN-γ-H2AX (+) might be associated with DSBs that arise during DNA replication.

**Figure 2:**
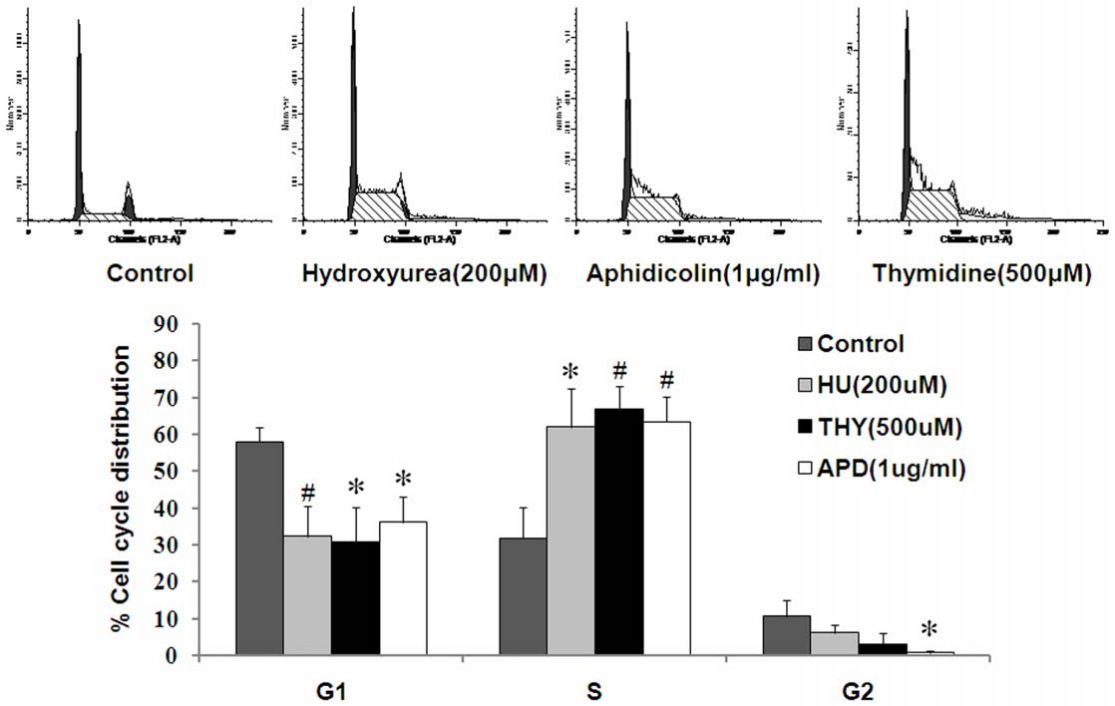
Hydroxyurea, aphidicolin and thymidine all cause S phase arrest. Cells were treated with different drugs for 24 h in all the panels. The distribution of cell cycle was assessed by flow cytometry. The experiments were performed in triplicate. Error bars represent standard deviation. *P<0.05, #*P*<0.01, when compared with control group.

To test that, we determined the frequency of MN-γ-H2AX (+) in MCF-7 cells that were treated with agents that cause replication stress. Hydroxyurea (HU), aphidicolin (APH) and thymidine (THY) are all well known to cause DNA replication stress and the phosphorylation of H2AX [27]–[30]. We chose the concentrations at which S phase arrest can be efficiently induced (Figure 2). After being treated with hydroxyurea for 24 h, and being released into drug-free fresh medium for 48 h, the cells were examined for the frequency of MN-γ-H2AX (+), as well as that of MN-γ-H2AX (−). Though the frequencies of both types of MN were increased, the elevation of MN-γ-H2AX (+) was more pronounced (Table 2). At 300 µM, the frequency of MN-γ-H2AX (+) reached 5.53×10^−2^, from 1.63×10^−2^ in the control (*P*<0.01). The relative proportion of MN-γ-H2AX (+) was increased to 36.13%, from 22.37% in the control. At all concentrations tested, the fold increase in the frequency of MN-γ-H2AX (−) was constantly lower than in that of MN-γ-H2AX (+).

**Table 2:** Induction of micronuclei by agents causing replication stress.

	No. Cells	No.MN	MN frequency (×10^−2^)	No.MN-	MN-γ-H2AX(+)	MN-γ-H2AX (+)		MN-γ-H2AX (−)	
				γ-H2AX(+)	/MN (%)	Frequency (×10^−2^)	Fold change over 0 µM	Frequency (×10^−2^)	Fold change over 0 µM
HU(0 µM)	1042	76	7.29	17	22.37	1.63	1.00	5.66	1.00
HU(100 µM)	1002	122	12.18**	34	27.87	3.39*	2.08	8.78**	1.55
HU(200 µM)	1001	133	13.29**	47	35.34	4.70**	2.88	8.59*	1.52
HU(300 µM)	1013	155	15.30**	56	36.13	5.53**	3.39	9.77**	1.73
APH(0 µg/ml)	1002	60	5.99	18	30.00	1.80	1.00	4.19	1.00
APH(0.5 µg/ml)	1013	99	9.77**	34	34.34	3.36*	1.87	6.42*	1.53
APH(1 µg/ml)	1002	134	13.37**	62	46.27	6.19**	3.45	7.19**	1.72
APH(2 µg/ml)	1022	141	13.80**	58	41.13	5.68**	3.16	8.12**	1.94
THY(0 µM)	1042	76	7.29	17	22.37	1.63	1.00	5.66	1.00
THY(300 µM)	1008	104	10.32*	35	33.65	3.47**	2.13	6.85	1.21
THY(600 µM)	1017	144	14.16**	51	35.42	5.01**	3.08	9.14**	1.62
THY(900 µM)	1000	150	15.00**	57	38.00	5.70**	3.50	9.30**	1.64

*P<0.05,

**P<0.01.

Aphidicolin was also shown to be more potent in inducing MN-γ-H2AX (+) than in inducing MN-γ-H2AX (−).While MN-γ-H2AX (+) only accounted for 30% of the total MN in control, they accounted for 46% in cells that were treated with 1 µg/ml for 24 h (Table 2).

Thymidine had a similar effect on the induction of MN. MN-γ-H2AX (+) were induced to a large extent than MN-γ-H2AX (−) in cells treated with thymidine (Table 2). The frequency of MN-γ-H2AX (+) increased by 3.5 fold at 900 µM, while that of MN-γ-H2AX (−) increased only by 1.6 fold.

Paclitaxel is known to impair the function of mitotic spindle apparatus by interfering with the breakdown of microtubules and induces MN [31], [32]. In cells that were treated with paclitaxel, the frequency of MN-γ-H2AX (+) remained relatively constant with increasing paclitaxel applied. The frequency of MN-γ-H2AX (−), on the other hand, showed a dose-dependent and statistically significant increase (Table 3). The results shown above indicated that the frequency of MN-γ-H2AX (+) can be efficiently elevated by the DNA replication stressors (hydroxyurea, aphidicolin and thymidine), but not by paclitaxel that does not cause DNA replication stress. Thus, unlike MN that are formed due to failures of chromosomes or chromosome fragments to segregate into daughter cells during anaphase, MN-γ-H2AX (+) formation probably involves active clustering and disposal of DNA DSBs before the onset of mitosis.

**Table 3:** Induction of micronuclei by paclitaxel.

	No. Cells	No.MN	MN frequency (×10^−2^)	No.MN-	MN-γ-H2AX(+)	MN-γ-H2AX (+)		MN-γ-H2AX (−)	
				γ-H2AX(+)	/MN (%)	Frequency (×10^−2^)	Fold change over 0 nM	Frequency (×10^−2^)	Fold change over 0 nM
0 nM	1002	64	6.39	14	21.88	1.40	1.00	4.99	1.00
5 nM	1000	94	9.40*	17	18.09	1.70	1.21	7.70*	1.54
10 nM	1009	132	13.08**	15	11.36	1.49	1.06	11.60**	2.32
15 nM	1004	155	15.44**	17	10.97	1.69	1.21	13.75**	2.75
20 nM	1003	158	15.75**	18	11.39	1.79	1.28	13.96**	2.80
30 nM	1003	142	14.16**	16	11.27	1.60	1.14	12.56**	2.52

*P<0.05,

**P<0.01.

### Generation of MN-γ-H2AX (+) during S phase

**Figure 3:**
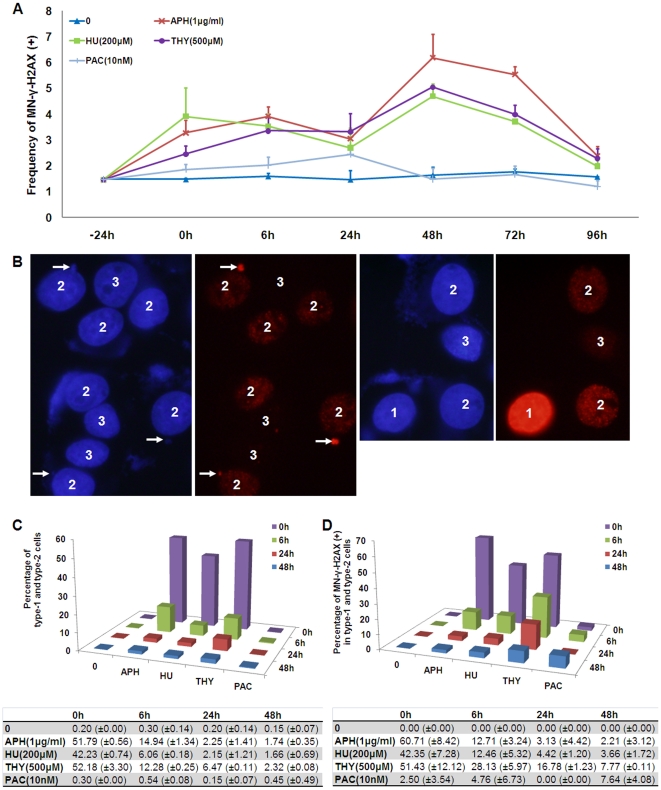
Newly formed MN-γ–H2AX (+) are associated with S phase cells experiencing replication stress. A. Changes in the frequency of MN-γ–H2AX (+) during the time course in MCF-7 cells treated with various agents. B. Distribution of γ–H2AX signals in cells experiencing replication stress. The cells were classified into three types, labeled as type 1, 2 and 3, by the distribution of γ–H2AX signals. MCF-7 cells were treated with thymidine (500 µM) for 24 h and were then fixed for immunofluorescence staining with antibody against γ-H2AX, as described in Materials and Methods. C. Decline in the percentage of cells exhibiting replication stress-induced DSBs over time. MCF-7 cells were treated with various agents at indicated concentrations for 24 h and were then processed and scored for the three types of cells. Percentages, together with their standard deviations, were also shown at the bottom. D. Decline in the association of MN-γ-H2AX with type 1 and type 2 cells over time. MCF-7 cells were treated with various agents at indicated concentrations for 24 h and were then processed and scored for MN-γ-H2AX. Percentages, together with their standard deviations, were also shown at the bottom.

To gain insight into the formation of MN-γ-H2AX (+) during an extended time course, we subjected cells to hydroxyurea, aphidicolin and thymidine, respectively, for 24 h and examined the frequencies of the MN-γ-H2AX (+) at different time points after the drugs were washed out. As shown in Figure 3A, the frequencies of MN-γ-H2AX (+) were found to be elevated at all time points till 72 h. They dropped at 96 h.

Sixty percent to 70% of the cells were in S phase at the end of the 24 h treatments, as shown by flow cytometry analysis (Figure 2). By the distribution of γ-H2AX signals in nuclei, the cells could be classified into three types: cells that were uniformly positive for γ-H2AX signals in their whole nuclei (type 1), cells that showed discrete γ-H2AX foci throughout the majority of a nucleus (type 2), and cells that had no or only occasional γ-H2AX foci (type 3) (Figure 3B). More than 99% of the untreated cells were of type 3. However, at the end of the treatment by each of the three agents (0 h), approximately half of the cells were of type 1 or type 2, which gradually decreased in proportion at 6 and 24 h after release from S phase arrest (Figure 3C). Considering that 60 to 70% of the cells were arrested in S phase at the end of each treatment (0 h), the concomitant surge of type 1 and type 2 cells indicated that these cells were positioned in S phase and were sustaining varying degree of replication stress-associated DNA damage. With the increasing clearance of DNA damage over time after release from drug treatment, the proportion of type 3 cells was increased at the expense of type 1 and type 2 cells. It should be noted that while all three agents induced the generation of type 1 cells, the type 1 cells were much rarer and almost dropped to basal level at 48 h (Figure 3C).

We then scored the MN-γ-H2AX (+) and their association with each of the three cell types at different time points after their release from treatments. As shown in Figure 3C & D, the newly generated MN-γ-H2AX (+), at 0 h, were exclusively associated with type 1 or type 2 cells, suggesting that they were formed during S phase. It appeared that while γ-H2AX signals were cleared in nuclei, due to the repair of the replication stress-induced DNA damage, they persisted in MN. As a consequence, type 2 cells hosting the newly generated MN-γ-H2AX (+) progressed to type 3 cells at later time points. Indeed, while DNA repair proteins can be recruited to DSB sites marked by γ-H2AX efficiently, they are usually absent in MN-γ-H2AX (+) [14]. Paclitaxel, on the other hand, had no effect on the induction of MN-γ-H2AX (+) at all time points examined, though it induced MN-γ-H2AX (−).

**Figure 4:**
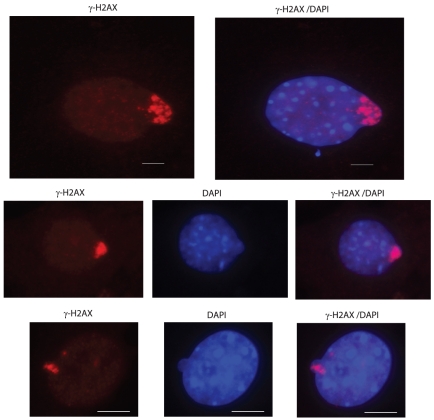
MN-γ-H2AX in formation. γ-H2AX signals are clustered in the nuclear blebs that may later form MN-γ-H2AX. Mouse skin fibroblasts at 5^th^ passage were processed for immunofluorescence staining with antibody against γ-H2AX. Bars, 5 µM.

MN-γ-H2AX (+) are much larger than individual γ-H2AX foci in nuclei. The drastic size difference between MN-γ-H2AX (+) and γ-H2AX foci suggests that the former may have been formed by an aggregation of multiple γ-H2AX foci, which each represents a DSB. We surveyed cells with nuclear projections or blebs that may later give rise to MN in mouse skin fibroblasts at 5^th^ passage. We encountered numerous instances in which γ-H2AX signals were being sequestered and were moving toward the tips (Figure 4). Such movements caught in snap shots probably reflect the steps leading to the formation of MN-γ-H2AX (+). It is likely that DSBs may become aggregated, before they are extruded from nucleus in clusters.

### Elevation of MN-γ-H2AX (+) in mutants with DNA replication defects

The findings above indicated that formation of MN-γ-H2AX (+) can be enhanced by agents that introduce DSBs in S phase, suggesting that such MN are derived from chromatin containing DSBs caused by replication stress. The findings prompted us to examine whether MN-γ-H2AX (+) would be enhanced in cells that have abnormalities in DNA replication or in S phase progression. We previously showed that RNA interference (RNAi) silencing of *CUL4B* led to a prolonged S phase and an increase in the frequency of micronuclei-containing HeLa cells [33]. We compared the frequency of MN-γ-H2AX (+) in *CUL4B* knockdown cells and in control cells. In three independent experiments, the relative proportion of MN-γ-H2AX (+) was consistently higher in *CUL4B* knockdown cells than in control. Combined, while only 19 of the 3037 control cells contained MN-γ-H2AX (+), as shown in Table 4, 41 of the 3182 *CUL4B* knockdown cells contained MN-γ-H2AX (+) (*P* = 0.008), indicating that MN-γ-H2AX (+) was associated with abnormalities in S phase progression.

**Table 4:** Elevation of MN-γ–H2AX (+) in miCUL4B cells.

	No. Cells	No.MN	MN frequency (×10^−2^)	No.MN-	MN-γ-H2AX(+)	MN-γ-H2AX (+)		MN-γ-H2AX (−)	
				γ-H2AX(+)	/MN (%)	Frequency (×10^−2^)	Fold change over miNeg	Frequency (×10^−2^)	Fold change over miNeg
miNeg-expt-1	1019	38	3.73	5	13.16	0.49	1.00	3.24	1.00
miCUL4B-expt-1	1005	44	4.38	12	27.27	1.19	2.43	3.18	0.98
miNeg-expt-2	1006	45	4.47	7	15.56	0.70	1.00	3.78	1.00
miCUL4B-expt-2	1172	66	5.63	14	21.21	1.19	1.71	4.44	1.17
miNeg-expt-3	1012	31	3.06	7	22.58	0.69	1.00	2.37	1.00
miCUL4B-expt-3	1005	47	4.68	15	31.91	1.49	2.15	3.18	1.34
miNeg-sum	3037	114	3.75	19	16.67	0.63	1.00	3.13	1.00
miCUL4B-sum	3182	157	4.93*	41	26.11	1.29**	2.06	3.65	1.17

*P<0.05,

**P<0.01.

**Figure 5:**
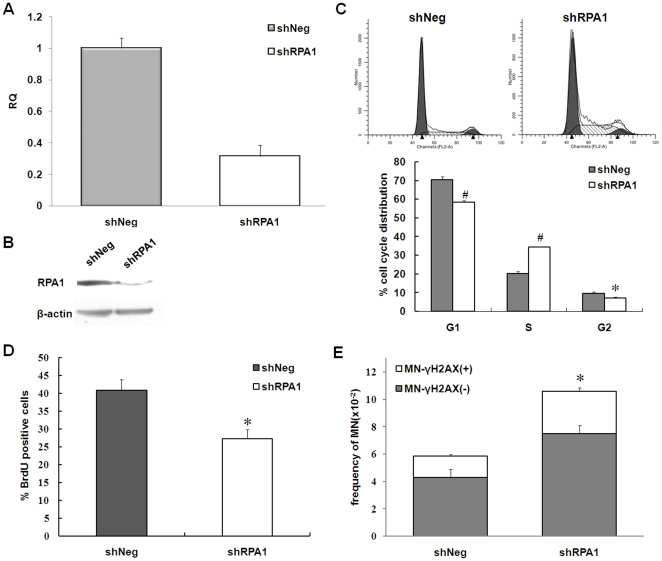
Increased MN formation in shRPA1 cells. A. Quantification of *RPA1* mRNA levels shRPA1 cells. RNA was extracted from shNeg MCF-7 cells and shRPA1 MCF-7 cells. The expression of RPA1 mRNA was quantified by real-time quantitative RT-PCR assay. The assay was performed in triplicate and relative means ± s.d. were shown. B. RPA1 protein level in shRPA1 cells. Equal amounts of protein lysates were subjected to SDS-PAGE (12%) and then detected using the antibodies against RPA1 and β-actin, respectively. C. Effect of RPA1 RNAi on cell cycle distribution of MCF-7 cells. The percentage of cells in S phase increased about 15% in shRPA1 cells when compared with shNeg cells (*P = 0.002). The experiments were performed in triplicate. Error bars represent standard deviation. For synchronization of cells, cells were starved in DMEM with 0.2% FBS for 48 h and then stimulated to initiate a new cell cycle in fresh media containing 10% FBS for 20 h. Afterward, cells were used for BrdU incorporation assay and cell cycle analysis. D. Reduction in cell proliferation as measured by BrdU incorporation assay. There was a marked reduction in BRDU-positive shRPA1 cells when compared with shNeg MCF-7 cells (*P = 0.004). The experiments were performed in triplicate. Error bars represent standard deviation. E. Increased MN-γ-H2AX (+) in shRPA1 cells. The assay was performed in triplicate and relative means ± s.d. were shown. *P = 0.008, when compared with shNeg MCF-7 cells.

RPA1 is one of the subunits of replication protein A, which is a trimeric complex composed of RPA1 (70-kDa), RPA2 (32-kDa), and RPA3 (14-kDa) [34]. Deficiency in RPA1 led to S phase arrest, increased level of DNA damage in S phase and increased chromosomal instability [35], [36]. We tested whether deficiency in RPA1 would affect the formation of MN-γ-H2AX (+). MCF-7 cells were transfected with RPA1-specific RNAi to suppress RPA1 expression, designated as shRPA1. Control cells were transfected with control oligonucleotides, designated as shNeg. The down-regulation of *RPA1* was evaluated by both real-time quantitative RT-PCR assay (Figure 5A) and Western blotting assay (Figure 5B). Results of real-time quantitative RT-PCR confirmed that the expression of RPA1 was reduced by more than 65% in shRPA1 cells as compared with shNeg cells (Figure 5A). As expected, cell cycle analysis showed that proportion of shRPA1 cells in S phase was higher than shNeg cells (*P*<0.01) (Figure 5C). Consistent with the distribution of cell cycles, shRPA1 cells exhibited a significant decrease in the percentage of BrdU positive cells compared with control cells, dropped from 40.86% in shNeg cells to 27.26% in shRPA1cells (*P*<0.01) (Figure 5D). We also found, as shown in Figure 5E, that shRPA1 cells exhibited a higher frequency of MN-γ-H2AX (+) when compared to control cells (P = 0.008). This finding provides additional evidence that MN-γ-H2AX (+) is associated with DNA replication stress.

## Discussion

By the presence or absence of uniform γ-H2AX marking of MN, we divided MN into MN-γ–H2AX (+) and MN-γ–H2AX (−). In a series of mammalian cells examined, we observed that MN-γ-H2AX (+) occurred at the frequency from less than 1×10^−2^ to 4×10^−2^ and accounted for 20% to 50% of total MN. We presented several lines of evidence suggesting that the MN-γ–H2AX (+) can be preferentially induced by replication stress. First, three agents that can cause S phase arrest all significantly induced the formation MN-γ–H2AX (+). The fold increase in frequency is more pronounced for MN-γ–H2AX (+) than for MN-γ–H2AX (−). As a consequence, the proportion of MN-γ–H2AX (+) relative to that of MN-γ–H2AX (−) was significantly increased in cells treated with agents that cause replication stress. Paclitaxel, which inhibits the disassembly of microtubules, only induced the formation of MN-γ–H2AX (−). Second, by examining the distribution of the two types of MN in cells at different stages of cell cycle, we showed that at MN-γ–H2AX (+) were formed during S phase. Third, in cells that experience a prolonged S phase due to the depletion of CUL4B by RNAi, only MN-γ–H2AX (+), but not MN-γ–H2AX (−), was significantly induced. Finally, in cells that were depleted of RPA1 by RNAi, and therefore were encountering DNA replication stress, the frequency of MN-γ–H2AX (+) is significantly increased.

Interestingly, hydroxyurea and aphidicolin also induce sister chromatid exchanges (SCE) and homologous recombination (HR) [37], [38]. While SCE and HR reflect the recombination repair of stalled replication forks, the induction of MN-γ–H2AX (+) by the same agents suggests that stalled or collapsed replication forks could also be disposed in the form of MN. This pathway may relieve a cell of persistent genotoxic stress imposed by irreparable DSBs, though it may result in deletions of genetic materials encapsulated in MN.

We observed the aggregation of DSBs in nuclear blebs, which indicates that MN-γ-H2AX (+) may be derived from the clustering of DNA DSBs inside nucleus. There are other possible routes by which the MN-γ-H2AX (+) may form. For example, MN-γ-H2AX (+) may originate from MN that contain a single γ-H2AX focus, which is then amplified to occupy a whole MN. We consider this unlikely because MN containing a single γ-H2AX focus were rarely observed in our experimental conditions. Another possibility is that MN-γ-H2AX (+) are derived from the whole or a segment of a chromosome. However, except for the sex bodies that are marked by γ-H2AX during spermatogenesis, there have been no cases in which H2AX can become phosphorylated along a whole chromosome in dividing cells.

Attention should be paid to whether the formation of MN-γ-H2AX (+) is associated with apoptosis. In HL-60 cells treated with topoisomerase I and II inhibitors, the activation of ATM and the phosphorylation of H2AX were shown to precede apoptosis [39]. We can not rule out the possibility that following the formation of MN-γ-H2AX (+) some cells may undergo apoptosis and produce additional MN. Even micronucleation induced by inhibitors of microtubules may trigger apoptosis of human lymphocytes [40]. The presence of MN-γ-H2AX (+) may thus reflect an early stage of apoptosis. However, we believe that the formation of MN-γ-H2AX (+) is not specifically associated with apoptosis. First, human and mouse fibroblasts usually undergo senescence instead of apoptosis, but MN-γ-H2AX (+) still forms in those types of cells (Table 1). Second, while paclitaxel is known to be a potent inducer of apoptosis in MCF-7 breast cancer cells, at concentrations enough to increase the frequency of MN-γ-H2AX (−) by at least two-fold, it had no effect on the frequency of MN-γ-H2AX (+) (Table 3). Importantly, cells with three or more MN were not included when we scored the MN in our experiments, which should have minimized possible interference by apoptotic events. Nevertheless, nuclear fragmentation associated with apoptosis was indeed shown to interfere with the scoring of MN and to lead to an overestimation of the genotoxic effect of tested substances [41].

It has been observed that different DSB-containing chromosome domains can be juxtaposed through clustering in G1 phase cells, possibly mediated by MRE11 [42]. Whatever the mechanism(s) are, the formation of MN-γ–H2AX (+) may involve an active process by which the broken DNA ends are tethered, clustered and then extruded into cytoplasm, in contrast to the passive process in which chromosome laggards or broken fragments are left behind during anaphase. Once a micronucleus is formed and lost during cell division, it becomes inevitable that the surviving cell will turn into a deletion mutant for the genetic materials encapsulated in the MN, unless such genetic materials are excess copies. It remains to be determined whether MN-γ–H2AX (+) represent excess genomic materials, or like other MN, they also lead to cellular genetic deletion.

It should be noted that while the replication stressors tended to preferentially induce MN-γ–H2AX (+), they also significantly induced MN-γ–H2AX (−). The induction of MN-γ–H2AX(−) is not unexpected considering that some previous studies showed that these replication stressors are all capable of causing chromosomal aberrations such as gaps, breaks and polyploidy [43]–[45]. RPA1-deficient cells also exhibit increased occurrence of chromosome breaks and aneuploidy [36]. Such chromosomal aberration may contribute to the increase of MN-γ–H2AX (−).

Formation of MN serves an indicator of intrinsic genomic instability and exposure to genotoxic agents [1], [2]. MN are traditionally divided into two types by the presence or absence of centromeres [3]. MN with centromeres, which can be induced by aneugens, are derived either from whole chromosomes, due to errors in mitotic apparatus. MN without centromeres, which can be induced by clastogens, are derived from broken chromosomes. Some mutagens can act both as an aneugen and as a clastogen. It is possible that some clastogens may actually induce MN via MN-γ–H2AX (+). It is expected that any agent that causes DNA replication stress would enhance MN-γ–H2AX (+), as hydroxyurea, aphidicolin and thymidine were shown to do in this report. Examination of MN-γ–H2AX (+) may provide a more accurate evaluation of the various genotoxic agents as well the genomic instability caused by defects in DNA replication and repair.
